# The knowledge and attitude concerning sport-related concussion among coaches: A survey study

**DOI:** 10.4102/sajp.v80i1.1955

**Published:** 2024-01-19

**Authors:** Thaer Manaseer, Saad M. Al-nassan, Akef M. Taifour

**Affiliations:** 1 Department of Sport Rehabilitation, Faculty of Physical Education and Sports Sciences, The Hashemite University, Zarqa, Jordan; 2 Department of Physical Therapy, Faculty of Allied Health Sciences, The Hashemite University, Zarqa, Jordan; 3 Department of Sport Management, Faculty of Physical Education and Sport Sciences, The Hashemite University, Zarqa, Jordan

**Keywords:** brain, brain concussion, athletes, schools, physical therapists

## Abstract

**Background:**

There are no studies investigating the level of knowledge about and attitude towards sports-related concussions (SRC) among sports coaches in Jordan.

**Objectives:**

This study aimed to examine the knowledge about and attitude towards SRC among Jordanian sports coaches.

**Method:**

Our study was based on a cross-sectional survey. An Arabic version of the questionnaire from the Centers for Disease Control and Prevention was used to collect data. The survey identified participants’ demographics and knowledge about (0–10 points with higher scores indicating a higher knowledge) and attitude towards (8–40 with lower scores indicating favourable attitudes) SRC. Descriptive statistics and the Kruskal–Wallis test were used to examine knowledge and attitude differences by demographic factors. Spearman’s correlation examined the correlation between the total knowledge and attitude scores.

**Results:**

Participants included 193 coaches (62 basketball, 66 martial arts, 30 soccer, and 35 swimming). The median total knowledge and attitude scores were 4 and 30, respectively. The total knowledge score was the highest in martial arts coaches (median = 4) and those with graduate degrees (median = 5). The total attitude score was the lowest among basketball coaches (median = 28) and those who were 40–50 years old (median = 28). No significant correlation between knowledge and attitude scores was observed.

**Conclusion:**

Jordanian coaches have a deficiency in knowledge about SRC and hold attitudes that are not consistent with current practice recommendations.

**Clinical implications:**

Knowledge and attitude about SRC can be improved through education, access to healthcare providers, and adherence to SRC management guidelines.

## Introduction

Participation in sports has positive effects on an individual’s physical health (Eime et al. 2013), mental health (Penedo & Dahn 2005), academic performance (Montecalbo-Ignacio, Iii & Buot 2017), and social skills (Eime et al. 2013). That said, participation in sports is associated with an increased risk of injuries. According to a study by the Centers for Disease Control and Prevention, approximately 8.6 million sports- and recreation-related injuries occur in the United States annually (Sheu, Chen & Hedegaard 2016). The severity of these injuries can range from minor bruises and sprains to more severe injuries such as cervical injuries (Swartz et al. 2009) and sports-related concussions (SRC) (McCrory et al. 2017).

Sports-related concussion is a type of mild traumatic brain injury that may result from a direct or indirect impact on the head (McCrory et al. 2017). Sports-related concussions may lead to significant short-term (e.g. impaired balance) (McCrory et al. 2017) and long-term (e.g. cognitive impairments) consequences (Manley et al. 2017). Overall, the exact epidemiology of SRC is difficult to determine because of the underreporting of this injury and varying definitions of the injury across the scientific literature (Voss et al. 2015). It has been estimated that from 1.6 m to 3.8 m SRCs occur in the United States annually (Harmon et al. 2013), with overall rates of SRC varying across levels of sports, sex, and different sports (Pierpoint & Collins 2021). The rate of SRC varies across sports and age, with college athletes having higher SRC rates than high school athletes (Pierpoint & Collins 2021). Across gender-comparable sports, females have higher SRC rates than males in both college and high school (Pierpoint & Collins 2021). That said, there is a need for continued monitoring of rates, patterns, and trends of SRC to better understand its rate and burden (Pierpoint & Collins 2021).

Given the seriousness of SRCs and possible short- and long-term impairments after injury, it is critical to provide all individuals involved in sports (i.e. athletic therapists, physiotherapists, physical education teachers, coaches, parents, and athletes) with the required education to identify SRCs when they occur. Providing such educational programmes, however, requires an understanding of the level of knowledge in SRCs among those individuals.

There is currently a limited number of studies examining the level of knowledge of SRCs among individuals involved in athletics in Jordan. A study examined the knowledge of SRCs among a sample of collegiate athletes in Jordan, the United States, and Ireland (Beidler et al. 2021). The study reported that collegiate athletes in Jordan demonstrated a lower level of knowledge of SRC compared with their peers in the United States. Furthermore, fewer Jordanian athletes reported receiving information on SRC than Irish or American athletes (Beidler et al. 2021). Hence, there is a need for further studies evaluating the level of knowledge of SRCs among specific populations of individuals involved in athletics in Jordan including, but not limited to, athletic therapists, physiotherapists, athletes, and coaches.

Access to healthcare providers on the sidelines during a sports event is generally limited (Pryor et al. 2015). Coaches are often the first to witness SRCs because they have direct contact with their athletes during practices and games (Register-Mihalik et al. 2013). Therefore, they need to have the necessary knowledge to recognise, respond to, and prevent SRCs (Parker et al. 2015; Register-Mihalik et al. 2013). Furthermore, sports coaches are expected to have SRC attitudes consistent with best practices for SRC safety (Sarmiento, Daugherty & DePadilla 2019). Hence, our study aimed to examine the knowledge about and attitude towards SRCs in a sample of Jordanian sports coaches.

## Methods

Our study was based on a cross-sectional survey and involved a convenient sample of Jordanian sports coaches who were available between December 2022 and March 2023 from various sports settings in Jordan. The total population size was not attainable given the absence of national records available to provide an exact count of sports coaches in Jordan. Participants were recruited from local sports coaching settings and through advertisements, social media, and word of mouth.

### The translation and adaptation of the questionnaire

The authors used an Arabic version of the previously validated pre-test questionnaire from the Centers for Disease Control and Prevention Concussion in Youth Sports campaign (Online Appendix 1). The original questionnaire involved three sections. Section one included questions related to participant demographics (i.e. the sports they were involved in, the age of the athletes they coached, the gender of the athletes they coached, the level of athletes they coached, and any prior training related to SRC). Section two involved 10 multiple choice questions that assess an individual’s knowledge about the mechanisms, signs, and symptoms of concussion, as well as return to play guidelines following concussions. Each question has four alternatives, and participants could choose one of them. Correct answers scored a value of 1, and incorrect responses scored a value of 0. This scoring approach resulted in a final score for each participant between 0 and 10 (Kirk et al. 2018). The overall score was used for analysis. Section three of the questionnaire included eight items related to coaches’ attitudes toward SRC. Each item was scored out of five (Likert scale). This section does not have a total score.

The authors translated the English version of the questionnaire into Arabic based on previously reported guidelines for forward and backward translation (Ozolins et al. 2020; Sousa & Rojjanasrirat 2011; World Health Organization 2011). Initially, two bilingual translators who have distinct backgrounds and whose mother language is Arabic forward translated the original survey from English to Arabic. This resulted in two Arabic copies of the survey. Next, a third independent translator compared the Arabic versions with the English version of the survey. A member of the research team resolved ambiguities and discrepancies in the Arabic version of the survey. Afterwards, a bilingual translator whose mother language is English, and who is knowledgeable about health terminology and concussion backward translated the Arabic survey into English. Finally, an expert and independent translator compared the Arabic version of the survey with the original English version in terms of the similarity of the instructions, items, and response format regarding wording, sentence structure, meaning, and relevance.

The face validity of the questionnaire used in our study was established through a panel review process. The panel consisted of the research team (three college professors with previous training in research methods, sports coaching, and/or SRC management and research) as well as two swimming coaches and one martial arts coach. The first author used the cognitive interviewing method (i.e. think-aloud) to examine the clarity and readability of the final Arabic version in a sample of nine sports coaches (Spark & Willis 2014). The first author documented participants’ feedback in writing.

### Data collection

#### Ethical considerations

Ethics approval (No: 4/2/2022/2023) was acquired from the Hashemite University Ethics Board. Completing the survey questions implied consent was given by the participants. All collected data were stored using one laptop. Only members of the research team had access to the laptop.

#### Participants

Coaches from both sexes were eligible to participate in our study, if they self-reported serving as coaches of a licensed sports coaching setting in the past 12 months or more. This investigation included sports with both high (e.g. soccer) and low (e.g. swimming) risks of concussion.

#### Procedures

All potential participants who expressed an interest and contacted the research team received a paper copy of the survey. All participants then completed the same survey and returned it to the research team. To minimise the risk of missing items, participants were required to answer all survey items before submission. Finally, a research assistant transferred the collected data from the survey to an Excel sheet for future analysis. The research assistant was fully blinded to the research question and hypothesis.

### Statistical analysis

Outcomes of interest included participants’ demographics, SRC knowledge, and attitudes towards SRC. Descriptive statistics (mean [standard deviation], median [range], or proportion as appropriate) were calculated and reported for all variables of interest. Participants’ demographics included age group, sex, level of education, number of years of experience, sex of athletes worked with (male, female, or both sexes), level of athletes worked with (beginner, developing, advanced, or multi-levels), whether a participant had to pull an athlete out of play because of a possible concussion (yes or no), whether a medical provider ever diagnosed an athlete a participant was coaching with a concussion (yes, no, or unsure), whether a healthcare provider attended practices or games (never, sometimes, or always), and whether a participant completed previous training on concussions (yes for one time, yes for more than one time, or never).

Knowledge was examined using 10 multiple-choice items. Correct answers scored one point and incorrect answers scored zero points. Resulting scores ranged from 0 to 10, with higher scores indicating better knowledge. Consistent with previous health knowledge studies (Bodson, Warner & Kepka 2016), the levels of knowledge were categorised as >80% (high), 50% – 80% (moderate), and <50% (low). Attitudes towards SRC were assessed using eight items on a five-point Likert scale. Item number three (concussions are less serious than other injuries) was reverse scored (i.e. 5 = 1 and 4 = 2) such that lower scores indicated better attitudes. Participants received one to five points per question for a potential score ranging from 8 to 40, with lower scores indicating better (i.e. favourable) attitudes.

Total scores for knowledge about SRC (out of 10) and attitudes toward SRC (out of 40) were used to examine between-group (i.e. coach age, coach sex, degree, coach experience, history of previous training in SRC, and sport) differences. The non-parametric Kruskal–Wallis test was performed to compare total scores related to knowledge about and attitudes towards SRC given the ordinal nature of these scores (Portney & Watkins 2009). Finally, a Spearman’s rho correlation test was conducted to examine the correlation between the knowledge about and attitudes towards SRC. The authors used SPSS Statistics 22 (IBM, Chicago, IL, USA) to analyse the data. The alpha level for statistical significance was set at *p* < 0.05.

## Results

### The translation and adaptation of the questionnaire

Before the forward translation of the English questionnaire, the research team agreed on deleting items related to the occupation and zip code as the questionnaire was directed to coaches, and no zip codes are used in Jordan. Instead, the research team agreed on adding a question related to the governate where coaches are used to coach. After the forward translation the research team, based on feedback from the translators, decided to add another alternative (i.e. I do not know) to all questions related to the knowledge of concussion to enhance knowledge reporting. The research team hypothesised that some of the participants would have no knowledge of concussions and, therefore, this alternative was added to minimise guessing. Finally, the independent translator decided that both the original and backward versions (Online Appendix 2) of the questionnaire were equivalent. Before pilot-testing the questionnaire, the research team agreed on adding questions related to participants’ age, sex, level of education, and number of years of experience to better understand the involved sample. The research team used the Arabic version of the questionnaire in Online Appendix 3. Pilot testing showed that the questionnaire was clear, and the questions were easy to read and follow as confirmed by participants.

#### Participants

All participants who received the survey (204 coaches) completed it (i.e. 100% response rate). Of those, 11 were not eligible as they self-reported serving as coaches of a licensed sports coaching setting for less than 12 months. The final sample included 193 (62 basketball, 66 martial arts, 30 soccer, and 35 swimming) coaches. Of those, 105 (54%) were 20–30 years old, 151 (78%) were males, 124 (68%) held a bachelor’s degree, 92 (48%) had more than 5 years of experience, 70 (36%) worked with athletes who were 14–18 years old, 133 (69%) coached athletes from both sexes, and 74 (38%) worked with experienced athletes. A total of158 coaches (82%) reported never pulling an athlete out of participation in sports because of a possible concussion. A total of 144 coaches (75%) reported that no medical provider ever diagnosed one of their athletes with a concussion. Seventy-one coaches (37%) reported that a healthcare provider always attends practices or games. In all,147 (76%) of the coaches reported not receiving training on concussions. Table 1 shows the detailed characteristics of the participants.

**TABLE 1:** Participants’ characteristics (*n* = 193).

Item	Basketball (*n* = 62)		Martial arts (*n* = 66)		Soccer (*n* = 30)		Swimming (*n* = 35)		Total (*n* = 193)	
	*n*	%	*n*	%	*n*	%	*n*	%	*n*	%
**Coach age group (years)**										
20–30	31	50	27	41	20	67	27	77	105	54
30–40	18	29	27	41	7	24	6	17	58	30
40–50	10	16	6	9	0	0	1	3	17	8
> 50	3	5	6	9	3	9	1	2	13	6
**Coach sex**										
Male	47	76	59	89	24	80	21	60	151	78
Female	15	24	7	11	6	20	14	40	42	22
**Degree**										
High school	11	18	22	33	3	10	7	20	43	22
Bachelor’s	41	66	32	49	25	84	26	74	124	64
Master’s	7	11	6	9	1	3	2	6	16	8
Doctor of Philosophy	3	4	6	9	1	3	0	0	10	5
**Experience in years**										
< 1	17	27	1	2	7	23	2	6	27	14
1– 3	13	21	17	26	8	27	16	46	54	28
3–5	8	13	47	70	5	17	6	17	66	34
> 5	24	39	1	2	10	33	11	31	46	24
**Athlete age group (years)**										
< 5	1	2	1	2	0	0	1	3	3	1
6–10	4	7	6	9	15	50	6	17	31	16
11–13	17	27	9	14	8	27	3	9	37	19
14–18	40	65	19	29	4	13	7	20	70	36
>18	0	0	31	47	3	10	18	51	52	27
**Coaches work with boys, girls, or both**										
Boys	19	30	11	17	17	56	0	0	47	24
Girls	7	11	1	2	2	7	3	9	13	6
Both	36	59	54	81	11	37	32	91	133	69
**The level of play of the athletes**										
Beginners	24	39	9	14	9	30	8	23	50	26
Developing	16	26	13	20	10	33	10	28	49	25
Advanced	4	7	5	8	2	7	7	23	18	9
Multi-levels	19	29	39	58	9	30	9	26	76	39
**A coach had to pull an athlete out of play because of a possible concussion**										
Yes	9	14	19	29	3	10	4	11	35	18
No	53	86	47	71	27	90	31	89	158	82
**A medical provider ever diagnosed an athlete a coach was coaching with a concussion**										
Yes	7	11	13	20	3	10	1	3	24	12
No	49	79	45	68	27	90	23	66	144	75
Unsure	6	10	8	12	0	0	11	31	25	13
**A healthcare provider attends our practices or games**										
Never	20	32	22	33	8	27	10	29	60	31
Sometimes	18	29	13	20	9	30	22	62	62	32
Always	24	39	31	47	13	43	3	9	71	37
**A coach has completed training on concussion**										
Yes (one time)	4	7	15	23	4	13	1	3	24	12
Yes (> one time)	5	8	13	20	1	3	0	0	19	10
Never	53	85	38	57	25	84	34	97	150	78

The chi-square analysis revealed significant associations between coach age and sport preference (χ²(9) = 20.695, *p* = 0.014), coach sex and sport preference (χ²(3) = 11.932, *p* = 0.008), and experience level and sport preference (χ²(12) = 62.343, *p* < 0.001). Younger coaches (20–30 years old) displayed a stronger preference for basketball and soccer, whereas coaches aged 30–40 years were less likely to prefer soccer. Male coaches showed preferences for martial arts, soccer, basketball, and swimming, while female coaches exhibited more varied sports preferences. Additionally, individuals tended to develop sports preferences as their experience level increased.

#### Knowledge of sports-related concussions

**FIGURE 1:**
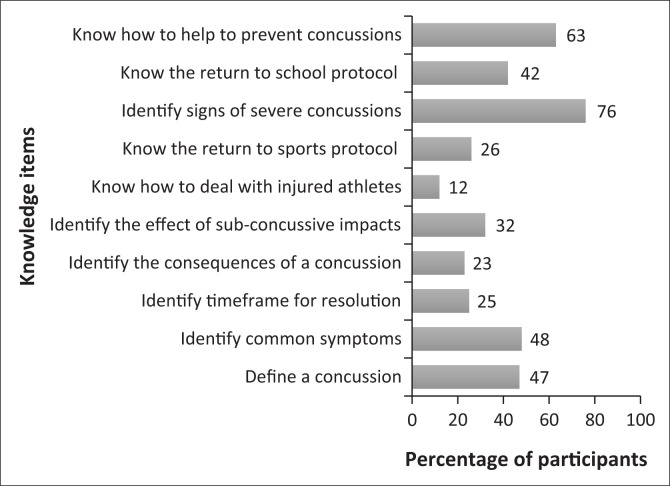
The percentage of correct answers among a sample of sports coaches (*n* = 193).

The authors calculated the Cronbach’s α for knowledge construct and it was 0.72. The median total knowledge score was 4 (ranging from 0 to 9) indicating a low level of knowledge about concussion. Overall, 115 (60%) participants demonstrated a low level of knowledge, 74 (38%) demonstrated a moderate level of knowledge, and 4 (2%) demonstrated a high level of knowledge about SRC. Figure 1 shows the percentage of correct answers for the whole sample. Table 2 shows the percentage of correct answers related to the section on knowledge of concussion stratified by groups.

**TABLE 2:** Percentage of correct group responses to the Centers for Disease Control and Prevention. Concussion in Youth Sport Questionnaire (*n* = 193).

Questionnaire item		Basketball (*n* = 62)		Martial Arts (*n* = 66)		Soccer (*n* = 30)		Swimming (*n* = 35)		Total (*n* = 193)	
		*n*	%	*n*	%	*n*	%	*n*	%	*n*	%
1	Coaches know the definition of a concussion	33	53	30	46	15	50	13	37	91	47
2	Coaches know the common symptoms of concussion.	39	63	32	49	9	30	12	34	92	48
3	Coaches know the timeframe for the resolution of concussion symptoms.	28	45	12	18	7	23	1	3	48	25
4	Coaches know the consequences of a previous concussion.	19	31	14	21	7	23	4	11	44	23
5	Coaches know the effect of sub-concussive head impacts.	12	19	31	47	9	30	10	29	62	32
6	Coaches know how to deal with an injured athlete who does not act right.	8	13	9	14	5	17	2	6	24	12
7	Coaches know how to return an injured athlete gradually to sports.	18	29	17	26	10	33	6	17	51	26
8	Coaches know signs of severe concussions that require emergency treatment.	48	77	56	84	19	63	23	66	146	76
9	Coaches know how to return an injured athlete gradually to school.	26	42	28	42	8	27	18	51	80	42
10	Coaches know how to help to prevent concussions.	32	52	48	73	16	53	26	74	122	63

Average score (%)		-	42	-	37	-	35	-	32	-	39

Table 3 shows knowledge scores stratified by sample characteristics. A statistically significant difference in knowledge about SRC was found across the four sports surveyed (χ² = 9.03, *df* = 3, *p* = 0.03). The total knowledge score was lower in swimming coaches (mean rank = 76) in comparison to soccer (mean rank = 86), basketball (mean rank = 107), and martial arts (mean rank = 104) coaches. Pairwise comparisons with Bonferroni adjustment revealed that swimming coaches had significantly lower knowledge scores than basketball coaches, *p* = 0.05. No significant differences were found in other sport pair comparisons (*p* > 0.05). The analysis also revealed a statistically significant difference in SRC knowledge based on the coaches’ educational degrees, χ² = 11.91, df = 3, *p* = 0.01. The total knowledge score was lower in coaches with high school education (mean rank = 76) in comparison to those with bachelor’s (mean rank = 99), master’s (125), and Doctor of Philosophy (mean rank = 118) degrees. Pairwise comparisons with Bonferroni correction indicated that coaches with high school education had lower knowledge scores than those with a bachelor’s (*p* = 0.019), Doctor of Philosophy (*p* = 0.032), and master’s degrees (*p* = 0.002). No significant differences existed among those with higher degrees (*p* > 0.05).

**TABLE 3:** Median knowledge and attitude score stratified by sample characteristic (*n* = 193).

Characteristics	Knowledge Total Score		Attitude Total Score	
	Median	Range	Median	Range
Sport * €				
Basketball	4.5	9	28.0	23
Martial arts	4.0	8	32.0	29
Soccer	3.5	8	30.0	28
Swimming	3.0	7	29.0	17
**Coach age group (years) €**				
20–30	4.0	9	29.0	28
30–40	4.0	8	30.0	28
40–50	5.0	6	28.0	29
> 50	3.5	4	37.5	15
**Coach sex**				
Male	4.0	9	30.0	29
Female	3.0	7	30.0	18
Degree *				
High school	3.0	5	30.0	29
Bachelor’s	4.0	9	29.5	28
Master’s	5.0	6	27.0	21
Doctor of Philosophy	5.0	7	29.0	15
**Experience in years**				
< 1	4.0	8	29.0	20
1–3	3.0	7	29.0	23
3–5	4.0	7	28.0	28
> 5	4.0	8	30.5	29
**Previous education in SRC**				
Yes (one time)	4.0	7	32.5	18
Yes (> one time)	5.0	6	30.0	24
Never	4.0	9	29.0	29

SRC, Sport-related Concussion.

* , Indicating significant between groups difference in total knowledge score (*p* < 0.05).

€, Indicating significant between groups difference in total attitude score (*p* < 0.05).

#### Attitude towards sports-related concussions

**FIGURE 2:**
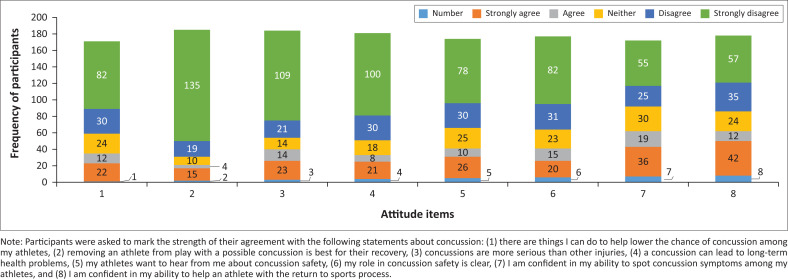
The frequency of responses to items related to the attitude of sports coaches towards sport-related concussions (*n* = 193).

The authors calculated the Cronbach’s α for the attitude construct and it was 0.65. The overall median attitude score was 30 (ranging from 11 to 40) indicating attitudes that are less consistent with current practice guidelines. Figure 2 shows the frequency of coaches’ responses to each attitude item. Table 4 shows the breakdown of coaches’ attitudes toward SRC stratified by groups.

**TABLE 4:** Coaches’ attitudes towards sport-related concussion by groups (basketball [*n* = 62], martial arts [*n* = 66], soccer [*n* = 30], swimming [*n* = 35]).

Item	1		2		3		4		5	
	*n*	%	*n*	%	*n*	%	*n*	%	*n*	%
**There are things I can do to help lower the chance of concussion among my athletes.**										
Total sample	22	11	12	6	47	24	30	16	82	43
Basketball	6	10	4	7	12	19	8	13	32	52
Martial arts	11	17	5	8	9	14	9	14	32	49
Soccer	4	13	3	10	11	37	3	10	9	30
Swimming	1	3	-	-	15	43	10	29	9	26
**Removing an athlete from play with a possible concussion is best for their recovery.**										
Total sample	15	8	4	2	20	10	10	10	135	70
Basketball	7	11	4	7	9	15	10	10	36	58
Martial arts	6	9	-	-	-	-	6	6	56	84
Soccer	1	3	-	-	3	10	20	20	20	67
Swimming	1	3	-	-	8	23	9	9	23	66
**Concussions are more serious than other injuries.**										
Total sample	23	12	14	7	26	14	21	11	109	57
Basketball	7	11	5	8	13	21	8	13	29	47
Martial arts	10	15	2	3	2	3	6	9	46	70
Soccer	3	10	1	3	5	17	3	10	18	60
Swimming	9	10	6	17	6	17	4	11	16	46
**A concussion can lead to long-term health problems.**										
Total sample	21	11	8	4	34	18	30	16	100	52
Basketball	3	5	4	7	15	24	12	19	28	45
Martial arts	19	20	2	3	6	9	5	8	40	61
Soccer	2	7	1	3	7	23	5	17	15	50
Swimming	3	9	1	3	6	17	8	23	17	49
**My athletes want to hear from me about concussion safety.**										
Total sample	26	14	10	5	49	25	30	16	78	40
Basketball	7	11	7	11	16	26	13	21	19	31
Martial arts	12	18	-	-	12	18	3	5	39	59
Soccer	7	23	1	3	7	23	6	20	9	30
Swimming	-	-	2	6	14	40	8	23	11	31
**My role in concussion safety is clear.**										
Total sample	20	10	15	8	45	23	31	16	82	43
Basketball	7	11	6	10	18	29	9	15	22	36
Martial arts	8	12	3	5	9	14	6	9	40	61
Soccer	4	13	5	17	3	10	6	20	12	40
Swimming	1	3	1	3	15	43	10	29	8	23
**I am confident in my ability to spot concussion symptoms among my athletes.**										
Total sample	36	18	19	10	58	30	25	13	55	29
Basketball	18	29	9	13	18	29	5	8	13	21
Martial arts	11	17	6	9	16	24	8	12	25	38
Soccer	6	20	3	10	6	20	4	13	11	37
Swimming	1	3	2	6	18	51	8	23	6	17
**I am confident in my ability to help an athlete with the return to sports process.**										
Total sample	42	22	12	6	47	24	35	18	57	30
Basketball	17	27	4	7	18	29	9	15	14	23
Martial arts	21	32	2	3	5	8	13	20	25	38
Soccer	4	13	4	13	8	27	3	10	11	37
Swimming	-	-	2	6	16	46	10	29	7	20

Note. For all items, ‘strongly agree’ = 1, ‘agree’ = 2, ‘neither agree nor disagree’ = 3, ‘disagree’ = 4 and ‘strongly disagree’ = 5.

Table 3 shows attitude scores stratified by sample characteristics. A statistically significant difference in attitudes towards SRC was evident across the four sports surveyed, χ² = 8.99, *df* = 3, p = 0.03. Martial arts coaches exhibited a higher attitude score (mean rank = 111) in comparison to soccer (mean rank = 94), swimming (mean rank = 99), and basketball (mean rank = 82) coaches. Pairwise comparisons with Bonferroni correction showed significant differences in attitude between martial arts and basketball coaches, *p* = 0.02. Furthermore, another statistically significant difference in attitudes towards SRC emerged based on the coaches’ age, χ² = 8.17, *df* = 3, *p* = 0.04. Coaches who were older than 50 years exhibited a higher attitude score (mean rank = 141) in comparison to those who were 20 to 30 (mean rank = 96), 30 to 40 (mean rank = 93), and 40 to 50 (mean rank = 87) years old. Pairwise comparisons with Bonferroni correction showed significant differences in attitude between the 40 and 50 age group and those older than 50 (*p* = 0.011), the 30–40 age group and those older than 50 (*p* = 0.007), and the 20–30 age group and those older than 50 (*p* = 0.008).

## Discussion

Access to healthcare providers on the sidelines during a sports event is generally limited (Pryor et al. 2015). Sports coaches are often the first to witness SRCs at a sports event (Register-Mihalik et al. 2013). Hence, they should possess a comprehensive understanding of SRC for the safety and welfare of their athletes. Currently, it is recommended that sports coaches gain knowledge pertaining to SRCs, including their definition, mechanisms, signs, and symptoms, as well as the proper management of SRC incidents and the return-to-play procedures post-injury (Feiss et al. 2020a; Glang et al. 2010). These recommendations are outlined comprehensively in the fact sheet provided by the Center for Disease Control’s HEADS UP education programme (Centers for Disease Control and Prevention 2019), a frequently utilised resource among sports coaches (Feiss et al. 2020a).

Our study aimed to examine the knowledge about and attitudes towards SRC in a sample of Jordanian sports coaches. Compared with current recommendations (Centers for Disease Control and Prevention 2019), the median total knowledge score was low (4 out of 10 [ranging from 0 to 9]) reflecting a deficiency in coaches’ general knowledge of SRC. Specifically, most of the coaches exhibited a lack of knowledge regarding the definition of SRC (*n* = 102, 53%), its common signs and symptoms (*n* = 102, 52%), the expected resolution timeframe (*n* = 144, 75%), its potential consequences (*n* = 148, 77%), the impact of sub-concussive forces (*n* = 131, 68%), SRC proper management (*n* = 170, 88%), the return-to-sport protocol (*n* = 143, 74%), and the return-to-school protocol (*n* = 102, 52%). The areas of greatest knowledge included recognising severe SRCs that require emergency treatment (*n* = 146, 76%) and knowing how to prevent SRCs (63%). A previous systematic review (Feiss et al. 2020b) reported a higher level of knowledge about SRC in sports coaches compared with our findings. This included their general knowledge of SRC, their ability to identify its common signs and symptoms, its management, as well as their knowledge of the return-to-play protocols following injury (Feiss et al. 2020b). Overall, our findings are similar to other studies (Broglio et al. 2010; Mrazik, Bawani & Krol 2011; McLeod et al. 2007; O’Donoghue et al. 2009; White et al. 2014) in terms of identifying gaps in knowledge about SRC among sports coaches.

Compared with current recommendations (Center for Disease Control and Prevention 2019), our findings also revealed that sports coaches in Jordan expressed attitudes that were not consistent with the current practice recommendations (i.e. the overall median attitude score was 30 [ranging from 11 to 40] with lower scores reflecting favourable attitudes towards SRC). For instance, few coaches agreed with the statements ‘There are things I can do to help lower the chance of concussion among my athletes’ (*n* = 34, 17%), ‘Removing an athlete from play with a possible concussion is best for their recovery’ (*n* = 19, 10%), ‘Concussions are more serious than other injuries’ (*n* = 37, 19%), ‘A concussion can lead to long-term health problems’ (*n* = 29, 15%), ‘My athletes want to hear from me about concussion safety’ (*n* = 36, 19%), ‘My role in concussion safety is clear’ (*n* = 35, 18%), ‘I am confident in my ability to spot concussion symptoms among my athletes’ (*n* = 55, 28%), and ‘I am confident in my ability to help an athlete with the return to sports process’ (*n* = 56, 28%). These findings contrasted to a study (Daugherty, DePadilla & Sarmiento 2019) involving a sample of 179 469 sports coaches, which reported that coaches held attitudes towards SRC that were more consistent with current practice recommendations. For instance, the majority of the coaches agreed with the statements ‘concussions are serious’ (*n* = 177 112, 99%), ‘I am confident in my ability to recognize concussion symptoms in youth athletes’ (*n* = 151 521, 84%), ‘there are things I can do to help prevent concussion among my athletes’ (*n* = 166 483, 95%), ‘I am confident in my ability to help an athlete with the return to play process’ (*n* = 143 307, 80%), and ‘plan to teach my athletes ways to prevent concussion’ (*n* = 167 426, 94%).

Data from our study revealed a non-significant correlation between the level of knowledge about and attitudes towards SRC. For instance, martial arts coaches had a higher level of knowledge about SRC, yet expressed attitudes that were less consistent with current practice recommendations in comparison to other sports. This finding is consistent with Kay et al. (2017) that assessed and compared the knowledge about and attitudes towards SRC in a sample of youth sports coaches. Our study concluded that ‘knowledge’ and ‘attitudes’ are different constructs that are not related. Hence, education programmes for coaches should address both the knowledge about and attitudes towards SRC (Kay et al. 2017).

We showed the deficiency in knowledge about and negative attitudes towards SRC among Jordanian sports coaches. This may compromise the safety of Jordanian athletes by potentially leading to delayed recognition, inadequate care, and suboptimal return-to-play decisions for athletes with SRC. Available recommendations to improve coaches’ knowledge about and attitudes towards SRC include the implementation of comprehensive SRC education programmes (Feiss et al. 2020a), increasing access to athletic therapists (Sarmiento et al. 2019), educating coaches on the importance of adhering to SRC management guidelines (Haran et al. 2016), and encouraging coaches to engage in ongoing reading of the relevant literature and staying informed about advancements in SRC management.

## Strengths and limitations

To the authors’ knowledge, this is the first study that examined the knowledge and attitude concerning SRC in a sample of Jordanian sports coaches. That said, our study has limitations. For instance, although the authors used published guidelines to translate the English version of the questionnaire from the Centre for Disease Control and Prevention Concussion in Youth Sports campaign, a risk of measurement bias remains. Future validation of the Arabic version of the questionnaire is warranted before widespread use in research. Furthermore, the sample size was small. This might lower the power of our study (Button et al. 2013). There is currently a need for studies to examine the actual number of coaches (actual population size) in Jordan to facilitate sample size calculation in future studies. Finally, the generalisability of the findings to female coaches is limited given that 78% of the coaches were males.

## Conclusion

Sports coaches in Jordan may have a deficiency in knowledge about SRC. Furthermore, they may hold attitudes that are not consistent with the current practice recommendations for the management of SRC. This may compromise the safety of Jordanian athletes by potentially leading to delayed recognition, inadequate care, and suboptimal return-to-play decisions for athletes with SRC. At this stage, the research team recommends the implementation of comprehensive SRC education programmes that target sports coaches in Jordan, increasing access to athletic therapists, educating coaches on the importance of adhering to SRC management guidelines, and encouraging coaches to engage in ongoing reading of the relevant literature and staying informed about advancements in SRC management.
